# Comparison of pregnancy outcomes and vaginal microbiota in women with endometriosis undergoing frozen embryo transfer using letrozole combined with HMG versus hormone replacement therapy with GnRH-a pretreatment

**DOI:** 10.7555/JBR.39.20250205

**Published:** 2026-07-28

**Authors:** Jie Zhang, Lei Dai, Chunyan Jiang, Yuxin Zhao, Xiang Ma, Yugui Cui, Jiayin Liu

**Affiliations:** State Key Laboratory of Reproductive Medicine and Offspring Health, Clinical Center of Reproductive Medicine, the First Affiliated Hospital of Nanjing Medical University, Nanjing, Jiangsu 210029, China

**Keywords:** endometriosis, letrozole, gonadotropin-releasing hormone agonist, frozen embryo transfer, vaginal microbiota

## Abstract

This study investigated differences in reproductive outcomes and vaginal microbiota profiles between two endometrial preparation protocols—letrozole (LE) combined with human menopausal gonadotropin (HMG) and hormone replacement therapy (HRT) with gonadotropin-releasing hormone agonist (GnRH-a) pretreatment—in women with endometriosis undergoing frozen embryo transfer (FET). Following 1∶1 propensity score matching, a total of 770 FET cycles were analyzed. No statistically significant differences were observed in live birth rates or clinical pregnancy rates between the two groups. However, the LE + HMG group showed a nonsignificant trend toward a lower miscarriage rate (13.7% *vs.* 19.8%, *P* = 0.070), as well as lower observed rates of cesarean delivery (64.9% *vs.* 75.4%, *P* = 0.020) and hypertensive disorders of pregnancy (4.8% *vs.* 10.1%, *P* = 0.039). Previous evidence suggests an association between GnRH-a treatment and reproductive tract microbiota composition. Given the clinical and ethical constraints of endometrial sampling during FET, vaginal microbiota was evaluated as a non-invasive indicator of lower reproductive tract microbial composition. In the prospective arm, vaginal samples from 55 women in the LE + HMG group and 50 in the GnRH-a HRT group were analyzed using 16S rRNA sequencing and droplet digital PCR. While no significant differences were observed in *Lactobacillus* or *Gardnerella* abundance, the GnRH-a HRT group showed higher relative abundances of several low-abundance taxa, such as *Escherichia-Shigella*, *Limosilactobacillus*, and *Staphylococcus*. In conclusion, although both protocols achieved comparable live birth outcomes, the LE + HMG regimen was associated with lower rates of cesarean delivery and hypertensive disorders of pregnancy, while the two protocols exhibited differences in several low-abundance vaginal bacterial taxa. However, longitudinal studies incorporating baseline and repeated sampling are needed to clarify temporal relationships and causality.

## Introduction

Endometriosis (EM) is a common, chronic, estrogen-dependent, and inflammatory disease characterized by the presence of endometrium-like tissue outside the uterus. It is estimated to affect 10%–15% of women of reproductive age^[1]^, with approximately one-third experiencing infertility^[2]^. Many of these women require *in vitro* fertilization (IVF) to achieve pregnancy^[3]^. In recent years, the global adoption of frozen-thawed embryo transfer (FET) has expanded rapidly, largely due to advancements in vitrification techniques and blastocyst culture.

A key determinant of FET success is the adequacy of endometrial preparation. As an estrogen-dependent condition, EM is characterized by abnormal aromatase overexpression in both ectopic lesions and the eutopic endometrium, leading to a disrupted estrogenic microenvironment^[4]^. This imbalance may interfere with embryo-endometrium communication, affecting pregnancy outcomes^[5]^. This unique pathological feature requires tailored endometrial preparation strategies for women with EM. However, the European Society of Human Reproduction and Embryology (ESHRE) guidelines offer no specific recommendations for endometrial preparation in women with EM^[3]^, rendering this an important area for investigation.

Gonadotropin-releasing hormone agonist (GnRH-a) has been shown to suppress local inflammation and reduce oxidative stress, thereby improving endometrial receptivity in women with EM^[6]^. Consequently, the hormone replacement therapy (HRT) with GnRH-a pretreatment protocol has become a common choice for FET in women with EM. However, the latest ESHRE guidelines no longer recommend the use of GnRH-a before assisted reproductive technology (ART) in EM patients^[3]^. Emerging evidence suggests that GnRH-a HRT does not offer significant advantages in improving fertility outcomes when compared with HRT alone or natural cycle (NC) protocols^[7–8]^. It is well known that both HRT and GnRH-a HRT protocols involve excessive supplementation of estradiol and progesterone, which may raise the risk of thromboembolic events^[9]^. Moreover, the lack of corpus luteum (CL) formation in these cycles has been associated with a higher risk of adverse maternal and perinatal outcomes^[10]^. Although NC protocols are considered safer and more physiological, they lack flexibility in scheduling, requiring frequent ovulation monitoring and facing higher cycle cancellation rates. These limitations underscore the need to explore alternative endometrial preparation strategies that are both effective and patient-friendly for women with EM undergoing FET.

Letrozole (LE), classified as a third-generation aromatase inhibitor, functions by suppressing estrogen synthesis and facilitating follicular development through negative feedback on the hypothalamic-pituitary axis^[11–12]^. Importantly, LE-induced ovulation results in the formation of a healthy CL, which reduces the risk of hypertensive disorders of pregnancy (HDP)^[13]^. To date, LE-based ovarian induction has been increasingly used for endometrial preparation in FET, especially for women with polycystic ovary syndrome^[14]^ and anovulation^[15]^. Beyond its reproductive applications, LE has also demonstrated efficacy in alleviating EM-related pain and reducing disease recurrence in both premenopausal and postmenopausal populations^[16–17]^. Emerging evidence suggests that LE may benefit the endometrium of women with EM by suppressing the estrogen-inflammatory axis^[18]^ and enhancing integrin αvβ3 expression, which could improve endometrial receptivity and implantation rates^[5,19]^. Despite its potential advantages, few studies have evaluated the effectiveness of LE-based ovarian induction in FET cycles for women with EM. Whether vaginal microbiota composition differs between women receiving different endometrial preparation protocols during FET remains largely unknown.

Recent microbiota research has revealed that treatment with GnRH-a for three to six months in women with EM for gynecological symptom management may lead to a marked decline in Lactobacillaceae and increased levels of Streptococcaceae, Staphylococcaceae, and Enterobacteriaceae in endometrial samples, suggesting a potential association between GnRH-a use and subclinical intrauterine infections^[20]^. Despite these findings, the impact of various endometrial preparation regimens on the reproductive tract microbiota during FET cycles remains poorly understood. Importantly, direct sampling of the endometrial microbiota on the day of embryo transfer is not feasible due to both ethical and clinical constraints—performing an endometrial biopsy at this critical time may damage the endometrium, reduce the likelihood of implantation, and raise ethical concerns regarding unnecessary harm. Interestingly, previous studies have demonstrated a continuous gradient in microbial composition extending from the vagina to the pelvic cavity^[21]^. Moreover, animal experiments have shown that transplantation of vaginal microbiota from patients with chronic endometritis into rats activates the endometrial Toll-like receptor 4 (TLR4)/nuclear factor kappa-light-chain-enhancer of activated B cells (NF-κB) pathway^[22]^, indicating that disturbances in the vaginal microbiota may reflect or induce similar changes in the upper reproductive tract. Vaginal microbiota was therefore evaluated as a non-invasive indicator of lower reproductive tract microbial composition, with the recognition that it cannot directly represent the endometrial microbiome.

Accordingly, this study was designed to evaluate and compare pregnancy and perinatal outcomes, as well as vaginal microbiota characteristics, in women with EM undergoing FET using either LE + human menopausal gonadotropin (HMG) or GnRH-a HRT protocols. By integrating clinical efficacy and microbial profiling, the present study aimed to provide evidence-based insights for optimizing endometrial preparation strategies in this unique patient population.

## Materials and methods

### Study design and sample collection

This retrospective cohort study included women with EM who underwent FET cycles with either LE + HMG or GnRH-a HRT protocol between January 2016 and December 2023. Inclusion criteria were as follows: (1) the first three eligible FET cycles per patient; (2) women aged < 43 years; (3) a single blastocyst transfer per cycle. Exclusion criteria included: (1) history of recurrent spontaneous abortion; (2) congenital uterine malformations; (3) use of preimplantation genetic testing; (4) loss to follow-up or incomplete data; (5) multiple pregnancies.

Additionally, a prospective study component involved vaginal samples collected from women with EM undergoing FET with either the LE + HMG (*n* = 55) or GnRH-a HRT (*n* = 50) protocol between January and June 2024. The inclusion criteria for the microbiota cohort included: (1) diagnosis of endometriosis; and (2) women aged < 43 years. However, embryo quality was not restricted, as the primary aim of this component was to investigate whether vaginal microbiota composition differed between the two endometrial preparation protocol groups, without addressing potential associations between microbiota alterations and pregnancy outcomes at this stage. The exclusion criteria were consistent with the retrospective cohort but included additional microbiota-specific considerations: patients were excluded if they had used antibiotics or vaginal probiotics within one month before sampling, or had acute reproductive tract infections, diabetes mellitus, or autoimmune diseases.

Vaginal secretions were collected before embryo transfer on the day of the procedure, before any procedural manipulation. A sterile speculum was used to expose the cervix, and two sterile cotton swabs were used to collect secretions from the upper third of the vaginal wall. One swab was used for 16S rRNA gene sequencing and the other for droplet digital PCR (ddPCR) analysis. The prospective microbiota cohort was recruited during a separate study period and did not overlap with the retrospective cohort. In total, 210 vaginal samples (two per patient) were collected. The study was approved by the ethics committee of the First Affiliated Hospital of Nanjing Medical University (No. 2023-SR-325).

### Diagnosis of EM and adenomyosis

EM was diagnosed either by surgical methods (laparoscopy or laparotomy) or by transvaginal ultrasound findings consistent with ovarian endometriotic cysts. The diagnosis of endometriotic cysts *via* ultrasound had to be documented in at least two separate menstrual cycles. Adenomyosis was diagnosed based on imaging criteria using transvaginal ultrasound, assessed by at least two highly skilled radiologists. The diagnosis was established when patients presented with clinical symptoms such as hypermenorrhea or dysmenorrhea and exhibited at least two of the following ultrasound features, as defined by the Morphological Uterus Sonographic Assessment (MUSA) criteria^[23]^.

### Endometrial preparation

**Figure 1:**
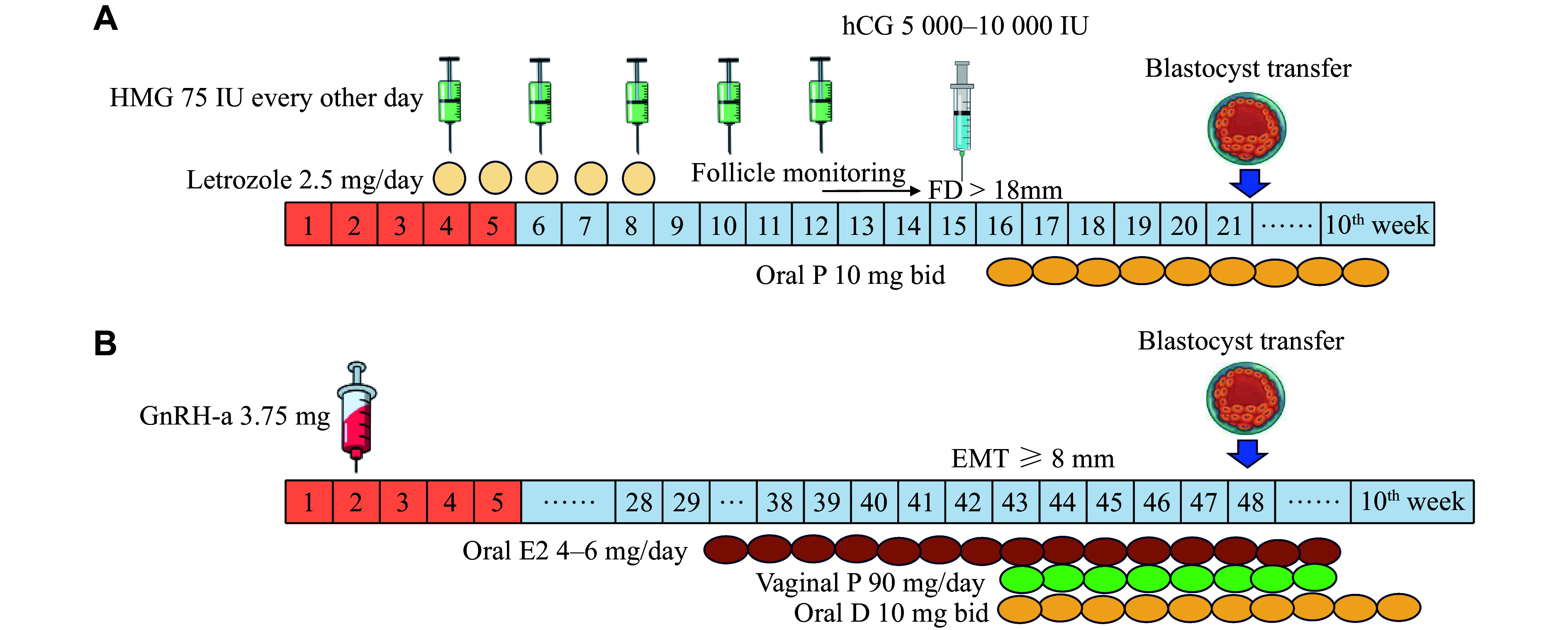
Schematic representation of protocols. A: Letrozole + HMG protocol. B: GnRH-a HRT protocol. Abbreviations: D, dydrogesterone; EMT, endometrial thickness; E2, estradiol; FD, follicle diameter; GnRH-a, gonadotropin-releasing hormone agonist; HMG, human menopausal gonadotropin; HRT, hormone replacement therapy.

In the LE + HMG group, LE (Hengrui, Lianyungang, China) was administered orally at a daily dose of 2.5 mg starting on the 4th day of the menstrual cycle for five consecutive days. Additionally, 75 IU of HMG (Lizhu, Zhuhai, China) was given every other day. Follicle monitoring began on the 12th day of the menstrual cycle and continued until the follicle diameter exceeded 18 mm. Subsequently, 5000–10000 IU of urinary human chorionic gonadotropin (hCG) was injected. Following triggering, oral dydrogesterone (Duphaston; Abbott Laboratories, Chicago, IL, USA) was prescribed at a dose of 10 mg twice daily for luteal phase support. On the 6th day after triggering, blastocyst transfer was performed, and Duphaston was continued until the 10th week of pregnancy (***Fig. 1A***).

In the GnRH-a HRT group, long-acting GnRH-a (Diphereline, 3.75 mg; Ipsen Pharma Biotech, Signes, France) was administered by intramuscular injection on menstrual cycle days 1–2. After 30 days, estradiol valerate (Progynova; Bayer, Leverkusen, North Rhine-Westphalia, Germany) was prescribed orally at a daily dose of 4–6 mg to stimulate endometrial proliferation until the endometrial thickness (EMT) reached ≥ 8 mm. Luteal support consisted of oral dydrogesterone (10 mg twice daily) combined with vaginal progesterone gel (Crinone, 90 mg once daily; Merck Serono, Darmstadt, Hesse, Germany). Blastocyst transfer was scheduled five days after initiating progesterone support. Upon confirmation of clinical pregnancy, estradiol and vaginal progesterone were tapered off by gestational weeks 7–8, while oral dydrogesterone was maintained until week 10 (***Fig. 1B***).

### Blastocyst morphological evaluation

Embryos were cultured to the blastocyst stage, typically achieved on day 5 or day 6 post-fertilization. Morphological assessment was performed according to the Gardner and Schoolcraft classification system. Embryos with a grade of 3BC or higher were deemed suitable for cryopreservation. Before frozen embryo transfer, thawed blastocysts were re-evaluated for structural integrity and developmental quality. High-quality blastocysts were defined as those graded AA, AB, BA, or BB with sufficient expansion, while those classified as AC, CA, BC, CB, or CC were considered of lower quality, despite meeting the minimum expansion criterion of grade 3.

### Data collection and outcome measures

Clinical characteristics of patients in this study were obtained from the institution's electronic database. Maternal and fetal outcome data were collected through telephone interviews with parents 1–3 months after the expected delivery date and recorded in the electronic medical records by trained nurses. The primary outcome was the live birth rate. Secondary outcomes included EMT on the transfer day, biochemical pregnancy rate, clinical pregnancy rate, miscarriage rate, and perinatal outcomes.

### 16S rRNA sequencing

Microbial genomic DNA was extracted from vaginal samples using the FastPure Stool DNA Isolation Kit (Vazyme, Nanjing, China). DNA concentration was measured with a NanoDrop 2000 spectrophotometer (Thermo Fisher Scientific, Waltham, MA, USA), and purity was verified by an A260/A280 ratio exceeding 1.8. PCR was performed using the primer pair 338F (5′-ACTCCTACGGGAGGCAGCA-3′) and 806R (5′-GGACTACHVGGGTWTCTAAT-3′). The amplification protocol consisted of an initial denaturation at 95 ℃ for 3 min, followed by 27 cycles of denaturation at 95 ℃ for 30 s, annealing at 55 ℃ for 30 s, and extension at 72 ℃ for 30 s, with a final elongation step at 72 ℃ for 10 min. PCR was carried out using a T100 Thermal Cycler (Bio-Rad, USA). Sequencing was conducted on the Illumina NextSeq 2000 PE300 platform. Raw paired-end reads were quality-filtered using fastp (version 0.19.6) and merged using FLASH (version 1.2.11). Reads shorter than 50 bp and sequences containing ambiguous bases were removed. Operational taxonomic units (OTUs) were clustered at 97% sequence similarity using USEARCH (version 11), with singleton and chimeric sequences removed during processing. Quality-controlled sequences were mapped to representative OTU sequences at a similarity threshold of ≥ 97% to generate the OTU abundance table. Sequences assigned to chloroplasts or mitochondria were excluded. Taxonomic assignment was performed using the RDP Classifier (version 2.13) against the SILVA 16S rRNA gene database (version 138), with a confidence threshold of 70%. To minimize the influence of unequal sequencing depth, all samples were rarefied to 20000 sequences before alpha- and beta-diversity analyses. No additional contaminant-deconvolution algorithm was applied.

### ddPCR quantification

Microbial genomic DNA was extracted using the MagicPure 32 Microbiome DNA Isolation Kit (Fullgene Biotech, Hangzhou, China). The *Lactobacillus* genus and *Gardnerella vaginalis* were detected based on 16S rRNA gene sequences. Target 16S rRNA gene sequences were retrieved from the National Center for Biotechnology Information (NCBI) database, and primers and probes were designed and validated for specificity using the NCBI BLAST tool. The primers and probes used for *Lactobacillus* were as follows: forward primer 338F (5*'*-AGAGGAGAGTGGAACTCCA-3*'*), reverse primer 806R (5*'*-CTCCCAACACTTAGCACT-3*'*), and probe 5*'*-FAM-CTGAGGCTCGAAAGCATGGGTAG-BHQ1-3*'*. For *Gardnerella* *vaginalis*, the following primers and probe were used: forward primer F (5*'*-GGTGAGTAATGCGTGACCAA-3*'*), reverse primer R (5*'*-GCCTACAAGCTGATAGGACG-3*'*), and probe P (5*'*-HEX-AATAGCTCTTGGAAACGGGTGG-BHQ1-3*'*). The ddPCR was performed with an initial denaturation at 95 ℃ for 5 min, followed by 40 cycles of 95 ℃ for 15 s and 58 ℃ for 25 s.

Fluorescent signals from each droplet were detected using the AccuONE Pro chip reader (Zhenuo Biotech Co., Ltd., Hangzhou, China). Fluorescence intensity was categorized using a threshold as "1" (positive) or "0" (negative). Target gene copy number was calculated using a Poisson distribution model.

### Statistical analysis

In the retrospective study, propensity score matching (PSM) was used to adjust for imbalanced covariates, including maternal age, infertility type, body mass index (BMI), type of ART, serum anti-Müllerian hormone (AMH) level, blastocyst quality, associated endometrioma, associated adenomyosis, and prior use of GnRH-a within 3 months. Propensity scores were estimated using logistic regression. LE + HMG cycles were matched 1∶1 with GnRH-a HRT cycles using nearest-neighbor matching without replacement and a caliper of 0.05 standard deviations of the logit of the propensity score. Covariate balance before and after matching was assessed using absolute standardized mean differences (SMDs), with values below 0.10 considered indicative of adequate balance. Propensity score distributions and empirical common support were also examined before and after matching.

To examine factors associated with live birth in women with EM undergoing FET, both univariate and multivariate logistic regression analyses were conducted after PSM. Before inclusion in the multivariate model, all variables were screened for multicollinearity. A backward stepwise elimination method was applied to identify significant factors independently associated with live birth. To evaluate the potential influence of repeated cycles contributed by the same woman, a sensitivity analysis was restricted to the first eligible FET cycle from each woman. Multivariable logistic regression was repeated in this patient-level cohort using the same candidate covariates as in the primary adjusted analysis.

Additionally, subgroup analyses were undertaken in specific subpopulations. Given that adenomyosis is a significant comorbidity of EM, the study population was stratified into patients with or without adenomyosis. Within each subgroup, maternal age, infertility type, BMI, type of ART, serum AMH level, blastocyst quality, associated endometrioma, and prior use of GnRH-a within 3 months were matched between the two groups.

For the microbiome analysis, genus-level comparisons were performed using the Wilcoxon rank-sum test. LEfSe analysis was conducted using an LDA score > 2.0 and a nominal *P* value < 0.05. No FDR correction was applied; therefore, taxon-level findings were considered exploratory. The consistency between ddPCR and 16S rRNA sequencing results was assessed using Bland-Altman plots and intraclass correlation coefficients (ICC). Bland-Altman analyses were performed using MedCalc software (version 15.6).

All statistical procedures were performed using SPSS version 26.0 (IBM Corp., Armonk, NY, USA) and R software version 4.4.1. For continuous variables, Student's *t*-test was applied when data followed a normal distribution, while the Mann–Whitney *U* test was used for non-normally distributed variables. Categorical data were analyzed using either the chi-square test or Fisher's exact test, as appropriate. For the clinical analyses, a two-sided *P* value < 0.05 was considered statistically significant. Microbiome taxon-level findings were interpreted as exploratory based on nominal *P* values.

## Results

### Baseline characteristics

**Figure 2:**
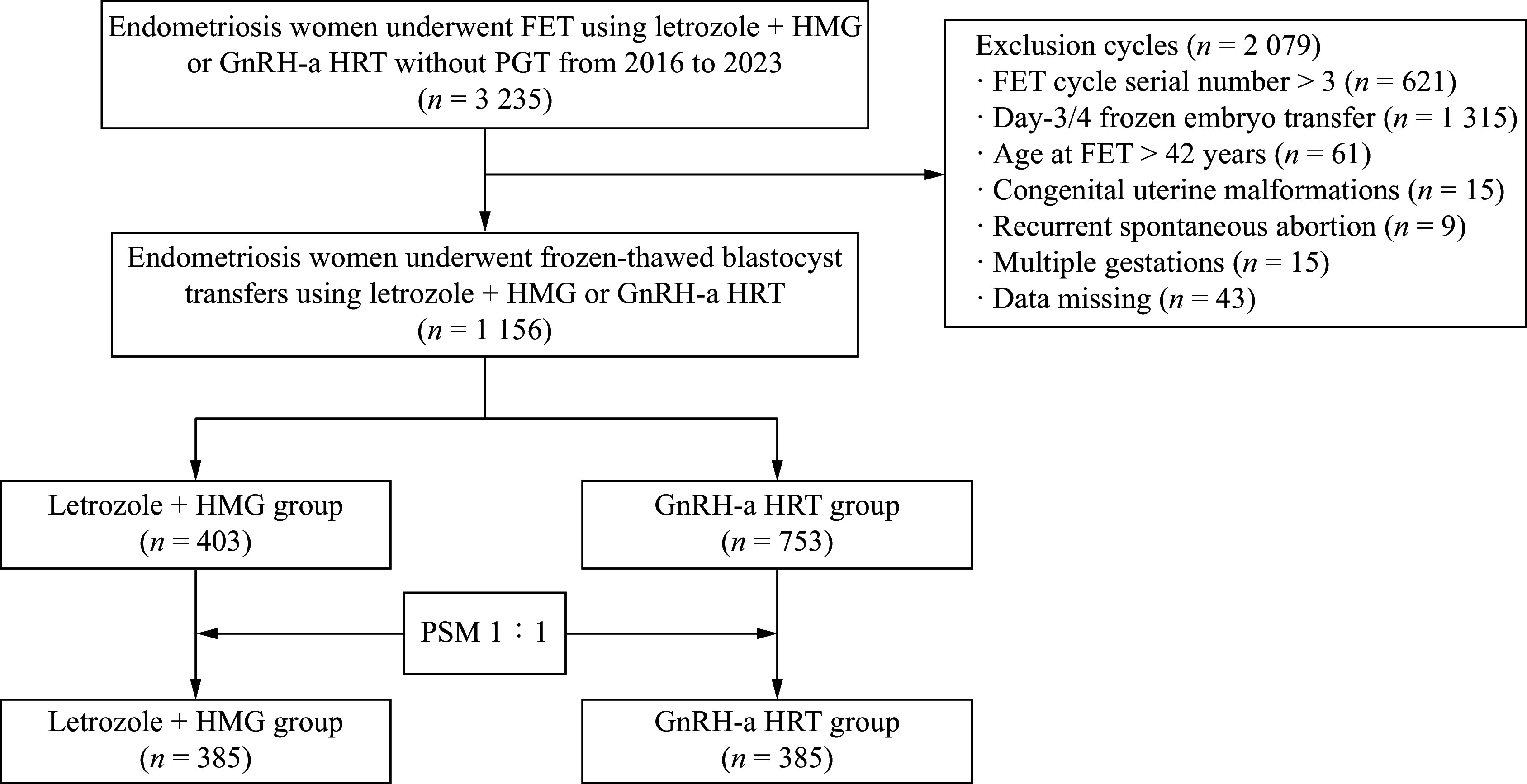
Flowchart of patient inclusion and exclusion criteria. Abbreviations: FET, frozen embryo transfer; GnRH-a, gonadotropin-releasing hormone agonist; HMG, human menopausal gonadotropin; HRT, hormone replacement therapy; PGT, preimplantation genetic testing; PSM, propensity score matching.

A total of 3235 cycles from patients with EM who underwent either LE + HMG or GnRH-a HRT cycles were screened. Of these, 1156 cycles from 948 patients (8 patients had 3 cycles, 192 had 2 cycles, and 748 had 1 cycle) were included. Ninety-eight women contributed cycles to both treatment groups. Exclusion reasons are shown in ***Fig. 2***. Among the 1156 cycles, 403 were from the LE + HMG group and 753 from the GnRH-a HRT group. After PSM at a 1∶1 ratio, 770 cycles from 663 women were retained, with 385 cycles in each group; 101 women contributed more than one matched cycle, 58 contributed cycles to both groups, and 14 of the 385 matched pairs comprised two cycles from the same woman.

Before PSM, serum AMH level, antral follicle count (AFC), associated endometrioma, adenomyosis, prior use of GnRH-a within 3 months, and blastocyst quality were different between the groups (***Table 1***). After PSM, all prespecified covariates were adequately balanced between the two groups, with SMDs below 0.10 and a maximum post-matching value of 0.055 (***Supplementary Table 1*** and ***Supplementary Fig. 1***). Propensity score distributions showed substantial overlap after matching (***Supplementary Table 2*** and ***Supplementary Fig. 2***).

**Table 1:** Baseline characteristics and assisted reproductive pregnancy outcomes between the two groups before and after PSM

Variables	Before PSM				After PSM		
	LE + HMG (*n* = 403)	GnRH-a HRT (*n* = 753)	*P* value		LE + HMG (*n* = 385)	GnRH-a HRT (*n* = 385)	*P* value
Maternal age at FET (years)	30.4 ± 3.6	30.8 ± 3.3	0.053		30.5 ± 3.6	30.7 ± 3.3	0.426
Paternal age at FET (years)	32.0 ± 3.8	31.7 ± 4.0	0.150		32.0 ± 3.9	31.7 ± 4.1	0.389
Duration of infertility (years)	3.6 ± 2.7	3.3 ± 2.5	0.067		3.6 ± 2.6	3.5 ± 2.6	0.530
BMI (kg/m^2^)	21.7 ± 2.7	21.5 ± 2.8	0.257		21.6 ± 2.7	21.7 ± 2.9	0.871
AMH (ng/mL)	6.0 ± 4.2	5.2 ± 3.9	0.001		5.8 ± 4.0	5.7 ± 3.9	0.825
Basal FSH level (IU/L)	6.9 (5.8–8.1)	7.1 (5.8–8.5)	0.104		6.9 (5.9–8.2)	6.9 (5.7–8.3)	0.848
AFC	16.0 ± 6.1	14.6 ± 6.2	< 0.001		15.9 ± 6.2	15.7 ± 6.3	0.686
Type of infertility [% (*n*)]			0.551				0.530
Primary	70.7 (285)	72.4 (545)			70.9 (273)	68.8 (265)	
Secondary	29.3 (118)	27.6 (208)			29.1 (112)	31.2 (120)	
Associated endometrioma [% (*n*)]	52.1 (210)	70.0 (527)	< 0.001		54.3 (209)	53.0 (204)	0.718
Associated adenomyosis [% (*n*)]	10.2 (41)	17.9 (135)	< 0.001		10.7 (41)	11.4 (44)	0.730
OS protocol [% (*n*)]			0.047				0.099
Agonist protocol	65.3 (263)	67.9 (511)			66.5 (256)	64.7 (249)	
Antagonist protocol	28.3 (114)	28.8 (217)			27.0 (104)	31.7 (122)	
Other protocols	6.4 (26)	3.3 (25)			6.5 (25)	3.6 (14)	
Type of ART [% (*n*)]			0.478				0.531
IVF	80.6 (325)	82.3 (620)			80.5 (310)	78.7 (303)	
ICSI	19.4 (78)	17.7 (133)			19.5 (75)	21.3 (82)	
No. of oocytes retrieved	11.9 ± 4.7	11.1 ± 4.4	0.003		11.7 ± 4.6	11.6 ± 4.5	0.807
No. of 2PN	9.8 ± 4.2	9.2 ± 4.0	0.024		9.6 ± 4.1	9.8 ± 4.1	0.604
Fertilization rate	0.8 ± 0.2	0.8 ± 0.2	0.335		0.8 ± 0.2	0.8 ± 0.2	0.189
Viable embryos	8.9 ± 3.9	8.3 ± 3.6	0.011		8.8 ± 3.8	8.8 ± 3.8	0.901
Blastocyst formation rate	0.6 ± 0.3	0.6 ± 0.2	0.081		0.6 ± 0.3	0.6 ± 0.2	0.279
Prior GnRH-a within 3 months [% (*n*)]	17.9 (72)	28.8 (217)	< 0.001		18.7 (72)	20.0 (77)	0.648
Good-quality blastocyst transfer [% (*n*)]	65.0 (262)	57.9 (436)	0.018		63.4 (244)	62.6 (241)	0.823
EMT on the transfer day (mm)	10.0 ± 1.7	9.9 ± 1.6	0.822		10.0 ± 1.7	9.9 ± 1.6	0.356
Biochemical pregnancy rate [% (*n*/*N*)]	67.7 (273/403)	73.3 (552/753)	0.046		67.0 (258/385)	75.6 (291/385)	0.009
Clinical pregnancy rate [% (*n*/*N*)]	63.3 (255/403)	65.7 (495/753)	0.403		62.6 (241/385)	67.0 (258/385)	0.200
Live birth rate [% (*n*/*N*)]	55.1 (222/403)	53.5 (403/753)	0.610		54.0 (208/385)	53.8 (207/385)	0.942
Miscarriage rate [% (*n*/*N*)]	12.9 (33/255)	18.6 (92/495)	0.049		13.7 (33/241)	19.8 (51/258)	0.070
Data are presented as mean ± standard deviation (SD) for normally distributed continuous variables, median (IQR) for non-normally distributed continuous variables, and % (*n*) or % (*n*/*N*) for categorical variables. *P* values were calculated using Student's *t*-test for normally distributed continuous variables, the Mann–Whitney *U* test for non-normally distributed continuous variables, and the *χ*² test or Fisher's exact test for categorical variables, as appropriate. Abbreviations: AFC, antral follicle count; AMH, anti-Müllerian hormone; ART, assisted reproductive technology; BMI, body mass index; EMT, endometrial thickness; FET, frozen embryo transfer; FSH, follicle-stimulating hormone; GnRH-a, gonadotropin-releasing hormone agonist; HMG, human menopausal gonadotropin; HRT, hormone replacement therapy; ICSI, intracytoplasmic sperm injection; IVF, *in vitro* fertilization; LE, letrozole; OS, ovarian stimulation; PSM, propensity score matching; 2PN, two-pronuclear zygotes.							

### Pregnancy and obstetric outcomes

As summarized in ***Table 1***, following PSM, live birth rate (54.0% *vs.* 53.8%, *P* = 0.942), clinical pregnancy rate (62.6% *vs.* 67.0%, *P* = 0.200), and EMT on the day of embryo transfer (10.0 mm *vs.* 9.9 mm, *P* = 0.356) were similar between the LE + HMG and GnRH-a HRT groups. Notably, the LE + HMG group demonstrated a significantly lower biochemical pregnancy rate (67.0% *vs.* 75.6%, *P* = 0.009) and a trend toward a reduced miscarriage rate (13.7% *vs.* 19.8%, *P* = 0.070). Moreover, patients in the LE + HMG group experienced fewer cesarean deliveries (64.9% *vs.* 75.4%, *P* = 0.020) and a lower incidence of HDP (4.8% *vs.* 10.1%, *P* = 0.039) (***Table 2***). In the sensitivity analysis restricted to the first eligible FET cycle from each woman, the endometrial preparation protocol remained unassociated with live birth after multivariable adjustment (adjusted OR 0.99, 95% CI 0.74–1.33; *P* = 0.946; ***Supplementary Table 3***), consistent with the primary findings. As shown in ***Table 3***, the duration of infertility, serum AMH level, associated adenomyosis, transfer of good-quality blastocysts, and EMT on transfer day were factors independently associated with live birth in women with EM undergoing FET.

**Table 2:** Perinatal outcomes between the two groups before and after PSM

Outcomes	Before PSM				After PSM		
	LE + HMG (*n* = 222)	GnRH-a HRT (*n* = 403)	*P* value		LE + HMG (*n* = 208)	GnRH-a HRT (*n* = 207)	*P* value
Gestational age (weeks)	38 (38–39)	39 (38–39)	0.459		38 (38–39)	39 (38–39)	0.185
Birth weight (g)	3410.4 ± 511.3	3412.3 ± 503.2	0.966		3404.9 ± 518.7	3435.7 ± 523.6	0.547
Delivery mode [% (*n*)]			< 0.001				0.020
Vaginal birth	35.6 (79)	21.6 (87)			35.1 (73)	24.6 (51)	
Cesarean section	64.4 (143)	78.4 (316)			64.9 (135)	75.4 (156)	
Newborn sex [% (*n*)]			0.849				0.404
Female	41.9 (93)	42.7 (172)			41.3 (86)	45.4 (94)	
Male	58.1 (129)	57.3 (231)			58.7 (122)	54.6 (113)	
LBW [% (*n*)]	3.2 (7)	4.2 (17)	0.507		3.4 (7)	5.3 (11)	0.330
Macrosomia [% (*n*)]	9.9 (22)	9.9 (40)	0.995		10.1 (21)	11.6 (24)	0.624
LGA [% (*n*)]	2.7 (6)	3.7 (15)	0.499		2.9 (6)	5.8 (12)	0.145
SGA [% (*n*)]	17.6 (39)	17.9 (72)	0.926		16.4 (34)	18.8 (39)	0.505
PTB [% (*n*)]	9.0 (20)	7.7 (31)	0.565		9.1 (19)	7.3 (15)	0.483
GDM [% (*n*)]	10.8 (24)	10.4 (42)	0.880		11.1 (23)	13.0 (27)	0.534
HDP [% (*n*)]	4.5 (10)	9.2 (37)	0.034		4.8 (10)	10.1 (21)	0.039
Placenta previa [% (*n*)]	3.2 (7)	5.5 (22)	0.190		3.4 (7)	3.4 (7)	0.993
Data are displayed as mean ± standard deviation (SD) or median (IQR) for continuous variables and % (*n*) for categorical variables. *P* values were calculated using Student's *t*-test for normally distributed continuous variables, the Mann–Whitney *U* test for non-normally distributed continuous variables, and the *χ*² test or Fisher's exact test for categorical variables, as appropriate. Abbreviations: GDM, gestational diabetes mellitus; GnRH-a, gonadotropin-releasing hormone agonist; HDP, hypertensive disorders of pregnancy; HMG, human menopausal gonadotropin; HRT, hormone replacement therapy; LBW, low birth weight; LE, letrozole; LGA, large for gestational age; PSM, propensity score matching; PTB, preterm birth; SGA, small for gestational age.							

**Table 3:** Univariate and multivariate logistic regression analyses of factors associated with live birth after PSM

Variables	Univariate analysis			Multivariate analysis	
	OR (95% CI)	*P* value		OR (95% CI)	*P* value
Maternal age at FET (years)	0.94 (0.90, 0.98)	0.002			
Paternal age at FET (years)	0.98 (0.95, 1.02)	0.359			
Duration of infertility (years)	0.95 (0.90, 1.01)	0.087		0.94 (0.88, 0.99)	0.019
BMI (kg/m^2^)	1.01 (0.96, 1.06)	0.680			
AMH (ng/mL)	1.07 (1.03, 1.11)	< 0.001		1.06 (1.02, 1.10)	0.003
Basal FSH level (IU/L)	1.00 (0.99, 1.02)	0.564			
AFC	1.02 (1.00, 1.05)	0.055			
Type of infertility (Secondary *vs.* Primary)	0.88 (0.65, 1.20)	0.427			
Associated endometrioma (Yes *vs.* No)	0.87 (0.66, 1.16)	0.339			
Associated adenomyosis (Yes *vs.* No)	0.38 (0.24, 0.61)	< 0.001		0.44 (0.27, 0.72)	0.001
Prior use of GnRH-a within 3 months (Yes *vs.* No)	0.84 (0.59, 1.20)	0.332			
Endometrial preparation protocol (GnRH-a *vs.* LE + HMG)	0.99 (0.75, 1.31)	0.942			
Good-quality blastocyst transfer (Yes *vs.* No)	1.95 (1.45, 2.62)	< 0.001		1.71 (1.26, 2.33)	0.001
EMT on the transfer day (mm)	1.12 (1.02, 1.22)	0.015		1.11 (1.03, 1.23)	0.013
Abbreviations: AFC, antral follicle count; AMH, anti-Müllerian hormone; BMI, body mass index; CI, confidence interval; EMT, endometrial thickness; FET, frozen embryo transfer; FSH, follicle-stimulating hormone; GnRH-a, gonadotropin-releasing hormone agonist; HMG, human menopausal gonadotropin; LE, letrozole; OR, odds ratio; PSM, propensity score matching.					

### Subgroup analysis

Adequate covariate balance was also achieved in the separately matched adenomyosis and non-adenomyosis subgroups, with all absolute SMDs below 0.10 after matching (***Supplementary Tables 4*** and ***5***). In an exploratory subgroup analysis of women with EM without adenomyosis, after PSM, 338 matched cycles were included in each group (***Supplementary Table 4***). The GnRH-a HRT group had a higher biochemical pregnancy rate (76.3% *vs.* 69.2%, *P* = 0.038), while the LE + HMG group had a lower miscarriage rate (10.9% *vs.* 17.8%, *P* = 0.037). In an exploratory subgroup of women with EM and adenomyosis, after PSM, 38 matched cycles from each group were included (***Supplementary Table 5***). Conversely, the GnRH-a HRT group had a higher live birth rate (42.1% *vs.* 21.1%, *P* = 0.048). Because of the limited sample size, these subgroup findings should be considered exploratory and require confirmation in larger cohorts.

### 16S rRNA sequencing and operational taxonomic unit (OTU) analysis

**Figure 3:**
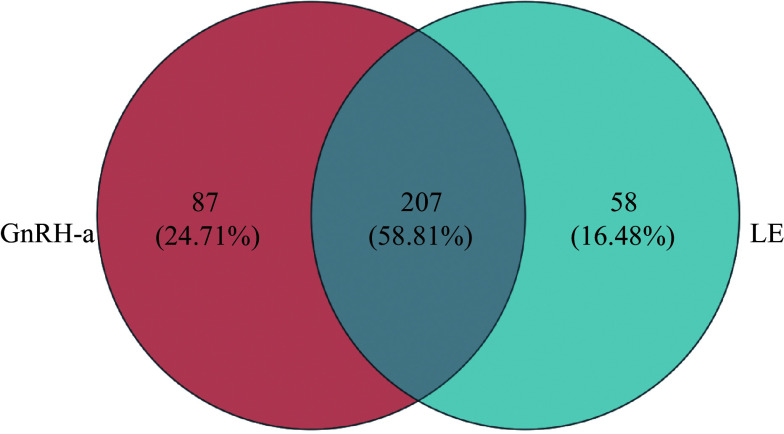
Venn diagram showing the shared and unique OTUs between the GnRH-a HRT group (GnRH-a) and the letrozole + HMG group (LE). Percentages indicate the proportion of OTUs relative to the total number of OTUs identified across all samples (*n* = 352). Abbreviations: GnRH-a, gonadotropin-releasing hormone agonist; HMG, human menopausal gonadotropin; HRT, hormone replacement therapy; LE, letrozole; OTUs, operational taxonomic units.

No statistically significant differences in the measured baseline characteristics were observed between the two groups (***Table 4***). After quality control and merging, 6097496 optimized sequences were retained for analysis. A total of 352 OTUs were identified across all samples, with 207 OTUs shared between the two groups. The LE + HMG group had 58 unique OTUs, while the GnRH-a HRT group had 87 unique OTUs, representing 16.48% and 24.71% of the total OTUs, respectively. Venn diagram analysis (***Fig. 3***) illustrates the overlap between the groups.

**Table 4:** Baseline characteristics of endometriosis patients for the investigation of the vaginal microbiota

Variables	LE + HMG (*n* = 55)	GnRH-a HRT (*n* = 50)	*P* value
Maternal age at FET (years)	33.1 ± 3.8	32.8 ± 3.9	0.705
Duration of infertility (years)	3.0 ± 2.7	3.3 ± 2.7	0.544
BMI (kg/m^2^)	21.8 ± 2.1	22.3 ± 3.0	0.312
AMH (ng/mL)	4.7 ± 4.1	3.8 ± 2.5	0.213
Basal FSH level (IU/L)	7.0 ± 2.2	7.7 ± 2.1	0.172
AFC	13.7 ± 5.7	14.2 ± 6.8	0.679
Primary infertility [% (*n*)]	65.5 (36)	52.0 (26)	0.161
Associated endometrioma [% (*n*)]	47.3 (26)	58.0 (29)	0.329
Associated adenomyosis [% (*n*)]	14.6 (8)	20.0 (10)	0.605
Data are presented as mean ± standard deviation (SD) for normally distributed continuous variables, and % (*n*) for categorical variables. *P* values were calculated using Student's *t*-test for continuous variables and the *χ*² test or Fisher's exact test for categorical variables, as appropriate.Abbreviations: AFC, antral follicle count; AMH, anti-Mullerian hormone; BMI, body mass index; FET, frozen embryo transfer; FSH, follicle-stimulating hormone; GnRH-a, gonadotropin-releasing hormone agonist; HMG, human menopausal gonadotropin; HRT, hormone replacement therapy; LE, letrozole.			

### Microbial diversity analysis

**Figure 4:**
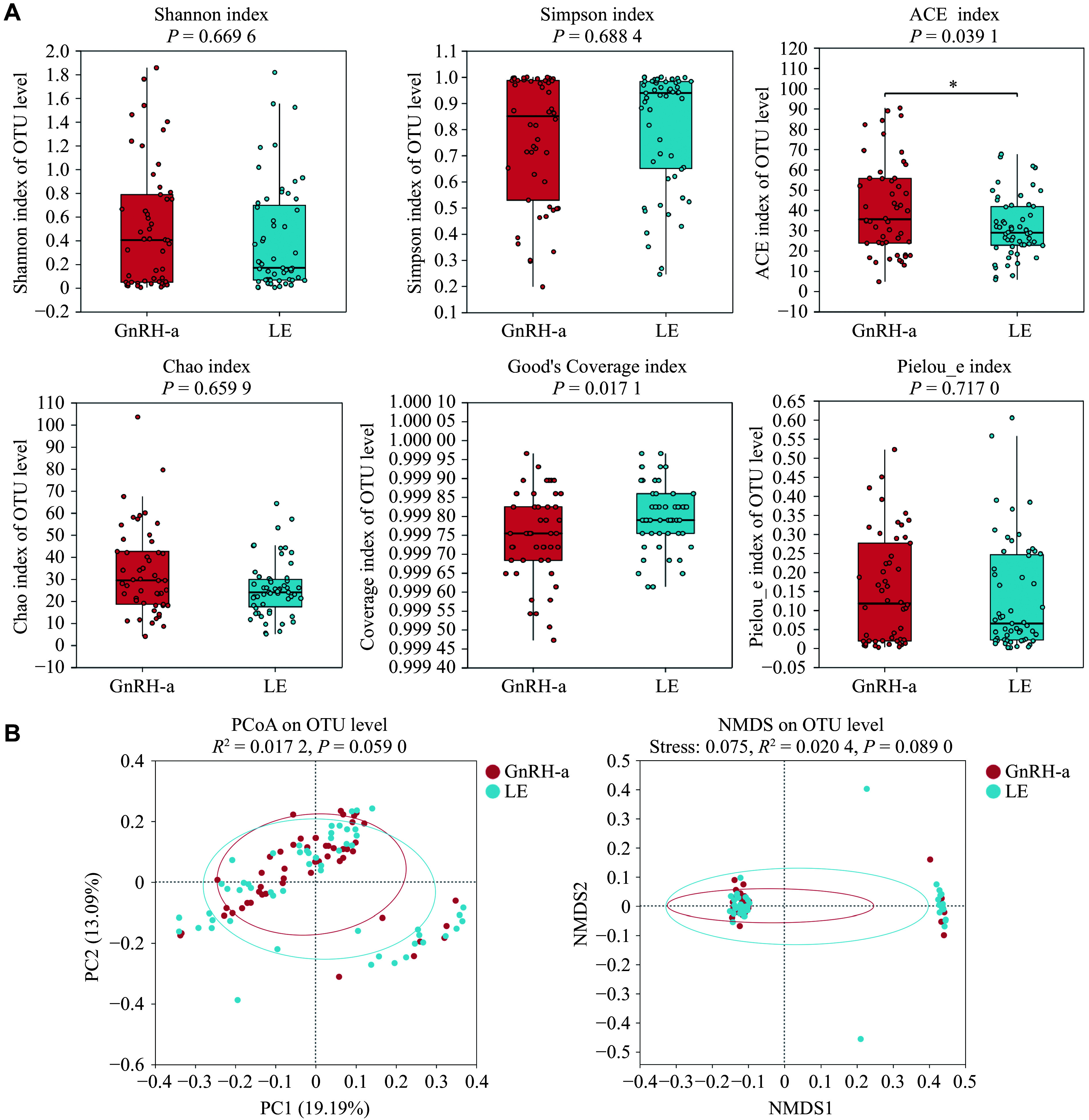
Comparison of vaginal microbiota diversity between the GnRH-a HRT (GnRH-a) and letrozole + HMG (LE) groups. A: Alpha diversity indices, including Shannon, Simpson, ACE, Chao, Good's Coverage, and Pielou_e, with group comparisons performed using the Mann–Whitney *U* test. B: Beta diversity analysis by principal coordinates analysis (PCoA) and non-metric multidimensional scaling (NMDS), with differences assessed using permutational multivariate analysis of variance (PERMANOVA). Data are from the GnRH-a (*n* = 50) and LE (*n* = 55) groups. Reported *P* values were nominal, and the ACE and Good's coverage findings were interpreted as exploratory. Abbreviations: ACE, abundance-based coverage estimator; GnRH-a, gonadotropin-releasing hormone agonist; HMG, human menopausal gonadotropin; HRT, hormone replacement therapy; OTU, operational taxonomic unit; Pielou_e, Pielou's evenness.

After rarefaction to 20000 sequences per sample, exploratory alpha-diversity analysis showed nominal between-group differences in the ACE index (*P* = 0.039) and Good's coverage index (*P* = 0.017) (***Fig. 4A***). Good's coverage values were close to 1 in both groups, indicating high sampling completeness rather than differences in sequencing depth. However, no significant differences were observed in the Shannon and Simpson diversity indices or community evenness (Pielou_e, *P* = 0.717). Beta diversity analysis, based on unweighted UniFrac distances, indicated no significant differences between the groups (PCoA, *P* = 0.059; NMDS, *P* = 0.089) (***Fig. 4B***).

### Microbial composition and differential abundance analysis

**Figure 5:**
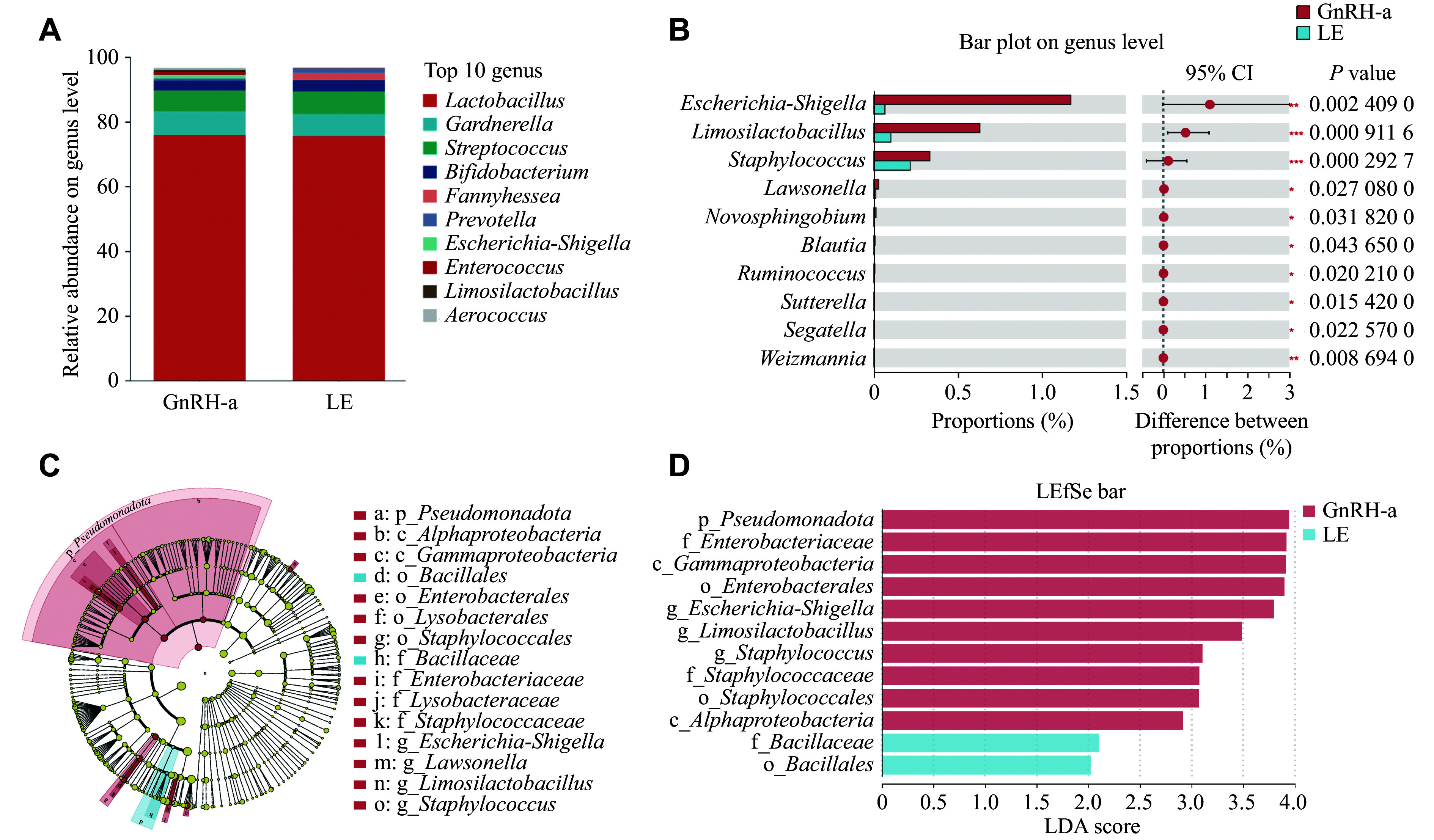
Comparison of vaginal microbiota composition between the GnRH-a HRT (GnRH-a) and letrozole + HMG (LE) groups. A: Bar plots depicting the relative abundance of the top 10 genera at the genus level. B: Genus-level microbial taxa showing nominal between-group differences in abundance between the two groups. C: Cladogram generated by linear discriminant analysis effect size (LEfSe) analysis. Each concentric circle represents a taxonomic level, from phylum (center) to class, order, family, and genus (outer layers). D: Linear discriminant analysis (LDA) score plot showing the effect size of taxa with significant intergroup differences. Reported *P* values were nominal and were not adjusted for multiple testing. Abbreviations: CI, confidence interval; GnRH-a, gonadotropin-releasing hormone agonist; HMG, human menopausal gonadotropin; HRT, hormone replacement therapy; LE, letrozole.

At the genus level, both groups were dominated by *Lactobacillus* (75.97% *vs.* 75.54%), *Gardnerella* (7.14% *vs.* 6.77%), and *Streptococcus* (6.57% *vs.* 6.98%) (***Fig. 5A***). However, among genera with abundance greater than 0.01%, levels of *Escherichia-Shigella* (1.17% *vs.* 0.06%, *P* < 0.01), *Limosilactobacillus* (0.63% *vs.* 0.10%, *P* < 0.01), and *Staphylococcus* (0.33% *vs.* 0.22%, *P* < 0.01) showed nominally higher relative abundances in the GnRH-a HRT group than the LE + HMG group (***Fig. 5B***).

### LEfSe differential analysis

An exploratory LEfSe analysis (LDA > 2) revealed that *Pseudomonadota taxa*, including *Gammaproteobacteria*, *Enterobacterales*, *Enterobacteriaceae*, and *Escherichia-Shigella*, were enriched in the GnRH-a HRT group (***Fig. 5C*** and ***5D***). Additionally, *Staphylococcaceae* and *Staphylococcus* were more abundant in this group. In contrast, the LE + HMG group exhibited nominally greater relative abundances of *Bacillaceae* and *Bacillales*.

### Detection of *Lactobacillus* and *Gardnerella* by ddPCR

**Figure 6:**
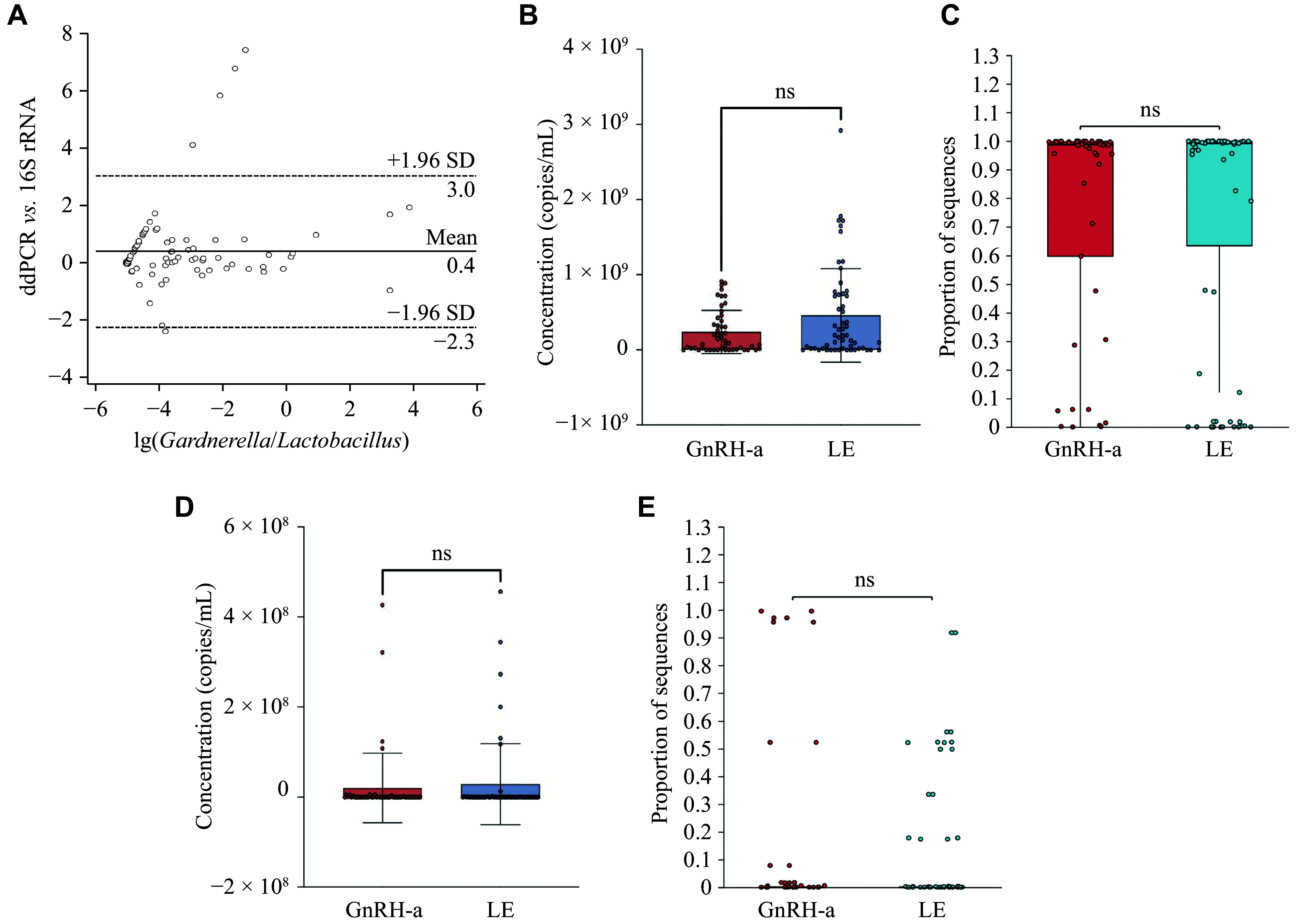
ddPCR and 16S rRNA sequencing in quantifying core vaginal microbiota. A: Bland–Altman plot demonstrating strong concordance between the two methods, with most data points falling within the 95% confidence interval; agreement was further assessed using intraclass correlation coefficient (ICC) analysis. B: Detection of *Lactobacillus* by ddPCR. C: Detection of *Lactobacillus* by 16S rRNA sequencing. D: Detection of *Gardnerella* by ddPCR. E: Detection of *Gardnerella* by 16S rRNA sequencing. Data are from the GnRH-a (*n* = 50) and LE (*n* = 55) groups. Comparisons between the GnRH-a and LE groups were performed using the Mann–Whitney *U* test. ^ns^*P* > 0.05.

*Lactobacillus* and *Gardnerella* rank among the most prevalent bacterial genera in the vaginal microbiota. In this study, we investigated the potential application of ddPCR in reproductive medicine by targeting these two genera. To evaluate the consistency between ddPCR and 16S rRNA sequencing, we log-transformed the copy numbers obtained from ddPCR and the relative abundances from 16S rRNA data for both *Lactobacillus* and *Gardnerella*. The ICC was 0.875 (95% confidence interval [CI]: 0.815–0.916), indicating excellent agreement (ICC ≥ 0.75), which was further supported by Bland-Altman analysis (***Fig. 6A***).

For *Lactobacillus*, the median copy number detected by ddPCR was 1.03 × 10^8^ (interquartile range [IQR]: 6.28 × 10^6^–3.97 × 10^8^) in the GnRH-a HRT group and 1.90 × 10^8^ (IQR: 1.78 × 10^7^–7.35 × 10^8^) in the LE + HMG group. The difference between the two groups was not statistically significant (*P* = 0.280) (***Fig. 6B***), a result consistent with 16S rRNA sequencing data (*P* = 0.601) (***Fig. 6C***).

For *Gardnerella*, ddPCR detected positive samples in 73.3% of cases versus 58.1% detected by 16S rRNA sequencing, suggesting that ddPCR may provide higher analytical sensitivity. The median *Gardnerella* copy number in the GnRH-a HRT group was 6.63 × 10^3^ (IQR: 0–5.75 × 10^5^), while in the LE + HMG group it was 2.81 × 10^4^ (IQR: 0–3.94 × 10^5^), with no significant intergroup difference (*P* = 0.393) (***Fig. 6D***). This finding was also consistent with 16S rRNA sequencing results, which showed similar relative abundances of *Gardnerella* between the two groups (*P* = 0.429) (***Fig. 6E***).

## Discussion

To our knowledge, this is the first large-scale study to compare pregnancy and perinatal outcomes between the LE + HMG and GnRH-a HRT groups in women with EM. To date, only two studies have attempted to evaluate different endometrial preparation protocols in this population. One study compared GnRH-a HRT, HRT, and NC protocols but did not include any LE-based regimens^[7]^. The other study incorporated LE + HMG in its comparison alongside GnRH-a HRT, HRT, and NC protocols^[8]^; however, it involved only 42 LE + HMG cycles, substantially limiting its statistical power and the generalizability of its findings.

The LE + HMG protocol was associated with lower rates of cesarean delivery and HDP and showed a nonsignificant trend toward a lower miscarriage rate. These findings may be partly explained by the presence or absence of the CL in different endometrial preparation protocols. In conventional HRT cycles, with or without GnRH-a pretreatment, the hypothalamic–pituitary–ovarian axis is suppressed, resulting in the absence of a functional CL. The CL plays a pivotal role in early pregnancy by producing not only estradiol and progesterone but also key vasoactive and angiogenic factors such as relaxin and vascular endothelial growth factor (VEGF). These hormones and signaling factors are essential for embryo implantation, endometrial decidualization, and proper placental development^[24]^. A deficiency in these factors may impair vascular remodeling and placental formation, although its potential contribution to the numerical difference in miscarriage rates remains speculative because this difference did not reach statistical significance. Moreover, relaxin is involved in cardiovascular adaptations during pregnancy; its absence has been implicated in the pathophysiology of preeclampsia and other hypertensive complications^[15]^. The LE + HMG protocol, by preserving ovulation and CL function through mild ovarian stimulation, ensures endogenous production of these crucial hormones. This may partly explain the lower observed incidence of HDP in the LE group. Nonetheless, further investigation is warranted to elucidate the association between HRT protocols and obstetric complications, such as miscarriage and HDP.

Blastocyst quality emerged as a factor independently associated with live birth in our analysis. Accumulating evidence suggests that impaired endometrial receptivity may not be the principal factor underlying implantation failure in ART, even in patients with EM^[25]^. Rather, embryo quality appears to play a more critical role in determining successful pregnancy outcomes^[26]^. Additionally, lower odds of live birth among women with EM, emphasizing the importance of considering adenomyosis as a significant prognostic factor. Its detrimental effect on reproductive outcomes may surpass that of EM alone^[27]^. In the exploratory subgroup analysis involving patients diagnosed with both EM and adenomyosis, those undergoing GnRH-a HRT demonstrated a significantly higher live birth rate compared with those treated with the LE + HMG protocol. This exploratory finding raises the possibility that GnRH-a pretreatment may be associated with improved live birth outcomes among women with concomitant adenomyosis^[28]^; however, the small sample size and multiple subgroup comparisons preclude definitive conclusions. The present subgroup finding requires confirmation in adequately powered prospective studies.

This study also compared vaginal microbiota composition and the abundances of key bacterial taxa between the two endometrial preparation protocol groups. The GnRH-a HRT group showed higher relative abundances of several low-abundance taxa, including *Escherichia-Shigella* and *Staphylococcus*. However, because microbiota was assessed at a single time point without pre-treatment or longitudinal sampling, these findings represent between-group associations and cannot be interpreted as protocol-induced microbial changes. One possible biological explanation for the observed differences may involve the hypoestrogenic state associated with GnRH-a treatment. Estrogen is known to play a crucial role in maintaining mucosal immunity by modulating the expression of antimicrobial peptides (AMPs), such as defensins and secretory leukocyte protease inhibitors, within the reproductive tract^[29–30]^. A reduction in estrogen levels may lead to decreased AMP expression, thereby compromising local defense mechanisms and facilitating colonization by opportunistic pathogens in the vaginal environment. However, this mechanism was not directly evaluated in the present study and should be regarded as a hypothesis requiring confirmation in longitudinal and mechanistic studies.

While 16S rRNA sequencing is widely used for qualitative analysis and to determine microbial diversity and relative abundance, it has limitations such as lower resolution, reduced detection efficiency for certain genera, and longer testing periods. Furthermore, the microbiota cohort was observational and relatively small, and the absence of statistically significant differences in measured baseline characteristics does not exclude residual or unmeasured confounding between the protocol groups. In contrast, ddPCR offers high sensitivity, specificity, and rapid diagnostic capabilities (within 3 h). In this study, we established a ddPCR assay for the detection of *Lactobacillus* and *Gardnerella* by designing specific primers and optimizing reaction conditions. The log-transformed *Gardnerella*-to-*Lactobacillus* ratios derived from ddPCR copy numbers and 16S rRNA relative abundances showed a high level of agreement, supporting the reliability of the ddPCR results and suggesting its potential clinical utility, which warrants further validation. Notably, ddPCR yielded a higher detection rate for *Gardnerella*, a low-abundance but potentially pathogenic bacterium that may be underrepresented in sequencing-based analyses, than 16S rRNA sequencing. This finding suggests a potential advantage of ddPCR for detecting low-abundance microorganisms within the reproductive tract.

This study has several limitations that should be acknowledged. First, it was a single-center, retrospective analysis, which may be susceptible to inherent selection biases. Prospective, multicenter randomized controlled trials are needed to validate our findings. Although some women contributed more than one FET cycle, a sensitivity analysis restricted to the first eligible cycle per woman yielded results consistent with the primary analysis. Nevertheless, the cycle-level PSM analysis did not explicitly account for within-patient correlation arising from repeated FET cycles, although the patient-level sensitivity analysis yielded consistent results. Second, NC and pure HRT protocols were not included due to their limited application at our center, which may restrict the generalizability of the results. Cesarean delivery and HDP were secondary exploratory outcomes. These findings remain susceptible to residual confounding and require confirmation in dedicated prospective studies. Additionally, perinatal outcomes were collected through telephone interviews, which may be less accurate than those obtained from standardized medical record reviews. Third, vaginal microbiota was assessed only once on the day of embryo transfer, without pretreatment or longitudinal sampling. Therefore, the observed between-group differences cannot be attributed causally to the endometrial preparation protocols. Vaginal samples provide information on lower reproductive tract microbial composition but cannot directly represent the endometrial microbiome. Taxon-level comparisons were exploratory and were not adjusted for multiple testing. An exploratory comparison between women with and without live birth was also limited by the small matched sample size and was therefore not presented in detail. Finally, the ddPCR analysis in this study was limited to *Lactobacillus* and *Gardnerella*, and did not include other potentially pathogenic taxa such as *Escherichia*, *Shigella*, *Staphylococcus*, *Streptococcus*, and *Enterococcus*.

In conclusion, our findings suggest that live birth rates were comparable between the LE + HMG and GnRH-a HRT protocols in women with EM undergoing FET. However, the LE + HMG group was associated with lower rates of cesarean delivery and hypertensive disorders of pregnancy. The two protocol groups exhibited differences in vaginal microbiota composition at the time of embryo transfer. As an exploratory methodological assessment, ddPCR showed promise as a rapid approach for quantifying selected vaginal microorganisms, although its clinical utility requires further validation. The observed differences in vaginal microbiota were associated with, rather than necessarily caused by, the endometrial preparation protocol. Longitudinal studies with serial sampling are needed to establish causality.

## Additional information

The online version contains supplementary materials available at http://www.jbr-pub.org.cn/article/doi/10.7555/JBR.39.20250205.
