# Supplementary material for: Comparison of pregnancy outcomes and vaginal microbiota in women with endometriosis undergoing frozen embryo transfer using letrozole combined with HMG versus hormone replacement therapy with GnRH-a pretreatment
Source: J Biomed Res. 2026 Jul 28;40(4):395–409. doi: 10.7555/JBR.39.20250205 (PMC13438300; doi:10.7555/JBR.39.20250205)
Supplement: Supplementary file 1 [file jbr-40-4-395-S1.pdf]

Available online at www.jbr-pub.org.cn

Open Access at PubMed Central

Journal of Biomedical Research, 2026 40(4): 1–5

*Supplementary Data*

JBR

# Comparison of pregnancy outcomes and vaginal microbiota in women with endometriosis undergoing frozen embryo transfer using letrozole combined with HMG versus hormone replacement therapy with GnRH-a pretreatment

Jie Zhang, Lei Dai, Chunyan Jiang, Yuxin Zhao, Xiang Ma, Yugui Cui✉, Jiayin Liu✉

*State Key Laboratory of Reproductive Medicine and Offspring Health, Clinical Center of Reproductive Medicine, The First Affiliated Hospital with Nanjing Medical University, Nanjing, Jiangsu 210029, China.*

***Supplementary Table 1*** **Covariate balance before and after propensity score matching**

| Covariate | Absolute SMD before matching | Absolute SMD after matching |
|---|---|---|
| Maternal age | 0.117 | 0.055 |
| BMI | 0.072 | 0.012 |
| AMH | 0.194 | 0.015 |
| Infertility type | 0.036 | 0.046 |
| Type of ART | 0.043 | 0.046 |
| Good-quality blastocyst transfer | 0.149 | 0.016 |
| Ovarian endometrioma | 0.358 | 0.026 |
| Adenomyosis | 0.257 | 0.026 |
| Prior GnRH-a within 3 months | 0.286 | 0.034 |

Absolute standardized mean differences (SMDs) were used to assess covariate balance before and after propensity score matching. An absolute SMD < 0.10 was considered indicative of adequate balance. All prespecified covariates achieved adequate balance after matching.
Abbreviations: AMH, anti-Müllerian hormone; ART, assisted reproductive technique; BMI, body mass index; GnRH-a, gonadotropin-releasing hormone agonist; SMD, standardized mean difference.

✉Corresponding authors: Jiayin Liu, E-mail: jyliu_nj@126.com, ORCID: 0000-0002-1472-4013; Yugui Cui, E-mail: cuiygnj@njmu.edu.cn, ORCID: 0000-0002-2866-6096.

Received: 10 May 2025; Revised: 28 May 2025; Accepted: 03 June 2025; Available online: 04 June 2025; Published date: 28 July 2026

CLC number: R711.71, Document code: A

The authors reported no conflicts of interest.

This is an open access article under the Creative Commons Attribution (CC BY 4.0) license, which permits others to distribute, remix, adapt and build upon this work, for commercial use, provided the original work is properly cited.

© 2026 by Journal of Biomedical Research.

https://doi.org/10.7555/JBR.39.20250205

***Supplementary Table 2*** **Propensity score overlap and matching diagnostics in the primary cohort**

| Diagnostic | LE + HMG | GnRH-a HRT | Overall |
|---|---|---|---|
| Cycles before matching (*n*) | 403 | 753 | 1 156 |
| Cycles retained after matching (*n*) | 385 | 385 | 770 |
| Cycles not retained after matching (*n*) | 18 | 368 | 386 |
| Proportion retained (%) | 95.5 | 51.1 | 66.6 |
| Matched pairs (*n*) | – | – | 385 |
| Overall propensity score range | – | – | 0.122–0.626 |
| Empirical common-support interval | – | – | 0.137–0.616 |
| Absolute within-pair propensity score difference, median (IQR) | – | – | 0.000 369 (0.000 138–0.001 897) |
| Absolute within-pair propensity score difference, range | – | – | 0–0.005 247 |
| Maximum absolute SMD after matching | – | – | 0.055 |

Propensity scores represented the estimated probability of receiving the LE + HMG protocol. The empirical common-support interval was defined as the overlap of the observed propensity score ranges in the two groups. Within-pair differences were calculated on the original propensity score scale. An absolute SMD < 0.10 indicated adequate covariate balance.
Abbreviations: IQR, interquartile range; SMD, standardized mean difference.

***Supplementary Table 3*** **Sensitivity analysis of factors associated with live birth restricted to the first eligible FET cycle per woman**

| Variables | Adjusted OR (95% CI) | *P* value |
|---|---|---|
| Endometrial preparation protocol, GnRH-a HRT *vs.* LE + HMG | 0.99 (0.74–1.33) | 0.946 |
| Maternal age at FET, per year | 0.96 (0.92–1.00) | 0.078 |
| Duration of infertility, per year | 0.96 (0.91–1.01) | 0.117 |
| BMI, per kg/m² | 1.00 (0.95–1.05) | 0.866 |
| AMH, per ng/mL | 1.04 (1.00–1.07) | 0.048 |
| Secondary *vs.* primary infertility | 0.78 (0.58–1.07) | 0.125 |
| ICSI *vs.* IVF | 0.75 (0.54–1.05) | 0.097 |
| Good-quality blastocyst transfer, yes *vs.* no | 1.59 (1.21–2.09) | 0.001 |
| Endometrioma, yes *vs.* no | 1.13 (0.84–1.53) | 0.417 |
| Adenomyosis, yes *vs.* no | 0.48 (0.32–0.70) | <0.001 |
| Prior use of GnRH-a within 3 months, yes *vs.* no | 0.85 (0.61–1.20) | 0.363 |
| Endometrial thickness, per mm | 1.07 (0.99–1.16) | 0.106 |

The analysis included the first eligible FET cycle from each of the 948 women. Odds ratios were estimated using a multivariable logistic regression model including the endometrial preparation protocol, maternal age at FET, duration of infertility, BMI, AMH, infertility type, assisted reproductive technique method, good-quality blastocyst transfer, endometrioma, adenomyosis, prior use of GnRH-a within 3 months, and endometrial thickness. An OR greater than 1 indicates higher odds of live birth.
Abbreviations: AMH, anti-Müllerian hormone; BMI, body mass index; CI, confidence interval; FET, frozen embryo transfer; GnRH-a, gonadotropin-releasing hormone agonist; HMG, human menopausal gonadotropin; HRT, hormone replacement therapy; ICSI, intracytoplasmic sperm injection; IVF, *in vitro* fertilization; LE, letrozole; OR, odds ratio.

***Supplementary Table 4*** **Baseline characteristics and pregnancy outcomes between the two groups of endometriosis women without adenomyosis before and after PSM**

| Variables | Before PSM | | | After PSM | | |
|---|---|---|---|---|---|---|
| | Letrozole + HMG (*n* = 362) | GnRH-a (*n* = 618) | *P* value | Letrozole + HMG (*n* = 338) | GnRH-a (*n* = 338) | *P* value |
| Maternal age at FET (years) | 30.3 ± 3.6 | 30.7 ± 3.3 | 0.147 | 30.4 ± 3.6 | 30.4 ± 3.20 | 0.955 |
| Paternal age at FET (years) | 32.0 ± 3.5 | 31.7 ± 4.4 | 0.148 | 31.9 ± 3.8 | 31.5 ± 4.1 | 0.206 |
| Duration of infertility (years) | 3.7 ± 2.7 | 3.4 ± 2.6 | 0.061 | 3.7 ± 2.8 | 3.5 ± 2.51 | 0.227 |
| BMI (kg/m$^2$) | 21.6 ± 2.7 | 21.3 ± 2.7 | 0.154 | 21.5 ± 2.7 | 21.3 ± 2.6 | 0.361 |
| AMH (ng/mL) | 6.1 ± 4.2 | 5.2 ± 3.7 | 0.001 | 5.8 ± 3.9 | 5.9 ± 4.0 | 0.847 |
| Basal FSH level (IU/L) | 6.9 (5.8–8.1) | 7.1 (5.8–8.6) | 0.074 | 7.0 (5.9–8.2) | 6.9 (5.7–8.3) | 0.962 |
| AFC | 16.3 ± 6.2 | 15.0 ± 6.3 | 0.002 | 16.0 ± 6.1 | 16.3 ± 6.7 | 0.622 |
| Type of infertility | | | 0.864 | | | 0.932 |
| Primary | 71.8 (260) | 72.3 (447) | | 71.3 (241) | 71.6 (242) | |
| Secondary | 28.2 (102) | 27.7 (171) | | 28.7 (97) | 28.4 (96) | |
| Associated endometrioma | 49.5 (179) | 67.3 (416) | <0.001 | 52.7 (178) | 52.7 (178) | 1.000 |
| OS protocol | | | 0.019 | | | 0.042 |
| Agonist protocol | 65.8 (238) | 69.7 (431) | | 66.3 (224) | 65.1 (220) | |
| Antagonist protocol | 27.6 (100) | 27.4 (169) | | 26.6 (90) | 31.7 (107) | |
| Other protocols | 6.6 (24) | 2.9 (18) | | 7.1 (24) | 3.2 (11) | |
| Type of ART | | | 0.536 | | | 0.230 |
| IVF | 80.1 (290) | 81.7 (505) | | 80.2 (271) | 83.7 (283) | |
| ICSI | 19.9 (72) | 18.3 (113) | | 19.8 (67) | 16.3 (55) | |
| No. of oocytes retrieved | 12.1 ± 4.7 | 11.3 ± 4.5 | 0.015 | 12.0 ± 4.7 | 11.9 ± 4.5 | 0.966 |
| No. of 2PN | 10.0 ± 4.2 | 9.5 ± 4.0 | 0.101 | 9.9 ± 4.2 | 9.9 ± 4.1 | 0.970 |
| Fertilization rate | 0.8 ± 0.2 | 0.8 ± 0.2 | 0.187 | 0.8 ± 0.2 | 0.8 ± 0.2 | 0.854 |
| Viable embryos | 9.1 ± 3.9 | 8.5 ± 3.7 | 0.021 | 9.0 ± 3.9 | 9.0 ± 3.8 | 0.826 |
| Blastocyst formation rate | 0.6 ± 0.3 | 0.6 ± 0.2 | 0.031 | 0.6 ± 0.3 | 0.6 ± 0.2 | 0.073 |
| Prior use of GnRH-a within 3 months | 17.4 (63) | 25.1 (155) | 0.005 | 18.3 (62) | 18.3 (62) | 1.000 |
| Good-quality blastocyst transfer | 68.8 (249) | 58.4 (361) | 0.001 | 66.9 (226) | 68.3 (231) | 0.681 |
| Outcomes | | | | | | |
| Endometrial thickness on the transfer day (mm) | 10.0 ± 1.7 | 10.0 ± 1.6 | 0.982 | 10.0 ± 1.7 | 10.1 ± 1.6 | 0.659 |
| Biochemical pregnancy rate [% (*n*/*N*)] | 70.4 (255/362) | 75.7 (468/618) | 0.069 | 69.2 (234/338) | 76.3 (258/338) | 0.038 |
| Clinical pregnancy rate [% (*n*/*N*)] | 66.3 (240/362) | 68.1 (421/618) | 0.556 | 65.1 (220/338) | 69.8 (236/338) | 0.189 |
| Live birth rate [% (*n*/*N*)] | 58.8 (213/362) | 56.2 (347/618) | 0.411 | 58.0 (196/338) | 57.4 (194/338) | 0.876 |
| Miscarriage rate [% (*n*/*N*)] | 11.3 (27/240) | 17.6 (74/421) | 0.030 | 10.9 (24/220) | 17.8 (42/236) | 0.037 |

Data are displayed as mean ± standard deviation (SD) or median (IQR) for continuous variables and % (*n*) for categorical variables. *P* values were calculated using Student's *t*-test for normally distributed continuous variables, the Mann–Whitney *U* test for non-normally distributed continuous variables, and the $\chi^2$ test or Fisher's exact test for categorical variables, as appropriate. Absolute standardized mean differences (SMDs) were used to assess the balance of prespecified matching covariates before and after PSM, with an absolute SMD < 0.10 indicating acceptable balance. All prespecified covariates had absolute SMDs < 0.10 after matching.
Abbreviations: 2PN, two-pronuclear zygotes; AFC, antral follicle count; AMH, anti-Mullerian hormone; ART, assisted reproductive technique; BMI, body mass index; FET, frozen embryo transfer; FSH, follicle-stimulating hormone; GnRH-a, gonadotropin-releasing hormone agonist; HMG, human menopausal gonadotropin; ICSI, intracytoplasmic sperm injection; IVF, *in vitro* fertilization; OS, ovarian stimulation; PSM, propensity score matching.

***Supplementary Table 5*** **Baseline characteristics and pregnancy outcomes between the two groups of women with endometriosis and adenomyosis before and after PSM**

| Variables | Before PSM | | | After PSM | | |
|---|---|---|---|---|---|---|
| | Letrozole + HMG (*n* = 41) | GnRH-a (*n* = 135) | *P* value | Letrozole + HMG (*n* = 38) | GnRH-a (*n* = 38) | *P* value |
| Maternal age at FET (years) | 30.8 ± 3.5 | 31.4 ± 3.3 | 0.339 | 30.9 ± 3.6 | 31.0 ± 3.1 | 0.892 |
| Paternal age at FET (years) | 31.8 ± 3.9 | 32.0 ± 3.8 | 0.827 | 31.9 ± 3.8 | 32.0 ± 3.3 | 0.899 |
| Duration of infertility (years) | 2.6 ± 2.0 | 3.0 ± 2.2 | 0.373 | 2.5 ± 1.7 | 3.2 ± 2.5 | 0.156 |
| BMI (kg/m$^2$) | 22.5 ± 2.5 | 22.2 ± 3.4 | 0.578 | 22.5 ± 2.6 | 22.7 ± 3.9 | 0.865 |
| AMH (ng/mL) | 3.5 (2.2–6.1) | 3.6 (2.3–6.4) | 0.654 | 3.6 (2.2–6.1) | 3.2 (2.0–5.5) | 0.593 |
| Basal FSH level (IU/L) | 6.5 (6.0–8.3) | 6.9 (5.9–8.2) | 0.948 | 6.7 (6.0–8.5) | 8.0 (6.0–8.5) | 0.403 |
| AFC | 14.1 ± 5.4 | 12.8 ± 5.7 | 0.189 | 14.4 ± 5.2 | 12.2 ± 6.0 | 0.086 |
| Type of infertility | | | 0.156 | | | 0.815 |
| Primary | 61.0 (25) | 72.6 (98) | | 57.9 (22) | 60.5 (23) | |
| Secondary | 39.0 (16) | 27.4 (37) | | 42.1 (16) | 39.5 (15) | |
| Associated endometrioma | 75.6 (31) | 82.2 (111) | 0.348 | 76.3 (29) | 79.0 (30) | 0.783 |
| OS protocol | | | 0.981 | | | 0.970 |
| Agonist protocol | 61.0 (25) | 59.3 (80) | | 63.1 (24) | 60.5 (23) | |
| Antagonist protocol | 34.1 (14) | 35.5 (48) | | 31.6 (12) | 34.2 (13) | |
| Other protocols | 4.9 (2) | 5.2 (7) | | 5.3 (2) | 5.3 (2) | |
| Type of ART | | | 0.977 | | | 0.744 |
| IVF | 85.4 (35) | 85.2 (115) | | 84.2 (32) | 86.8 (33) | |
| ICSI | 14.6 (6) | 14.8 (20) | | 15.8 (6) | 13.2 (5) | |
| No. of oocytes retrieved | 10.2 ± 4.1 | 9.7 ± 4.1 | 0.578 | 10.2 ± 4.3 | 10.2 ± 3.9 | 0.978 |
| No. of 2PN | 8.3 ± 3.6 | 7.9 ± 3.4 | 0.536 | 8.4 ± 3.7 | 8.2 ± 3.1 | 0.789 |
| Fertilization rate | 0.8 ± 0.2 | 0.8 ± 0.1 | 0.635 | 0.8 ± 0.2 | 0.8 ± 0.1 | 0.583 |
| Viable embryos | 7.2 ± 2.8 | 7.4 ± 3.2 | 0.681 | 7.2 ± 2.9 | 7.5 ± 2.8 | 0.634 |
| Blastocyst formation rate | 0.6 ± 0.3 | 0.6 ± 0.3 | 0.741 | 0.6 ± 0.3 | 0.6 ± 0.2 | 0.703 |
| Prior GnRH-a within 3 months | 22.0 (9) | 45.9 (62) | 0.006 | 23.7 (9) | 21.1 (8) | 0.783 |
| Good-quality blastocyst transfer | 31.7 (13) | 55.6 (75) | 0.007 | 34.2 (13) | 36.8 (14) | 0.811 |
| Outcomes | | | | | | |
| Endometrial thickness on the transfer day (mm) | 9.7 ± 1.9 | 9.7 ± 1.6 | 0.946 | 9.6 ± 1.4 | 10.0 ± 1.8 | 0.279 |
| Biochemical pregnancy rate [% (*n*/*N*)] | 43.9 (18/41) | 62.2 (84/135) | 0.037 | 44.7 (17/38) | 63.2 (24/38) | 0.107 |
| Clinical pregnancy rate [% (*n*/*N*)] | 36.6 (15/41) | 54.8 (74/135) | 0.041 | 36.8 (14/38) | 55.3 (21/38) | 0.107 |
| Live birth rate [% (*n*/*N*)] | 22.0 (9/41) | 41.5 (56/135) | 0.023 | 21.1 (8/38) | 42.1 (16/38) | 0.048 |
| Miscarriage rate [% (*n*/*N*)] | 40.0 (6/15) | 24.3 (18/74) | 0.212 | 42.9 (6/14) | 23.8 (5/21) | 0.234 |

Data are displayed as mean ± standard deviation (SD) or median (IQR) for continuous variables and as % (*n*) or % (*n*/*N*) for categorical variables. *P* values were calculated using Student's *t*-test for normally distributed continuous variables, the Mann–Whitney *U* test for non-normally distributed continuous variables, and the $\chi^2$ test or Fisher's exact test for categorical variables, as appropriate. Absolute standardized mean differences (SMDs) were used to assess the balance of prespecified matching covariates before and after PSM, with an absolute SMD < 0.10 indicating acceptable balance. All prespecified covariates had absolute SMDs < 0.10 after matching.

Abbreviations: 2PN, two-pronuclear zygotes; AFC, antral follicle count; AMH, anti-Mullerian hormone; ART, assisted reproductive technique; BMI, body mass index; FET, frozen embryo transfer; FSH, follicle-stimulating hormone; GnRH-a, gonadotropin-releasing hormone agonist; HMG, human menopausal gonadotropin; ICSI, intracytoplasmic sperm injection; IVF, *in vitro* fertilization; OS, ovarian stimulation; PSM, propensity score matching.

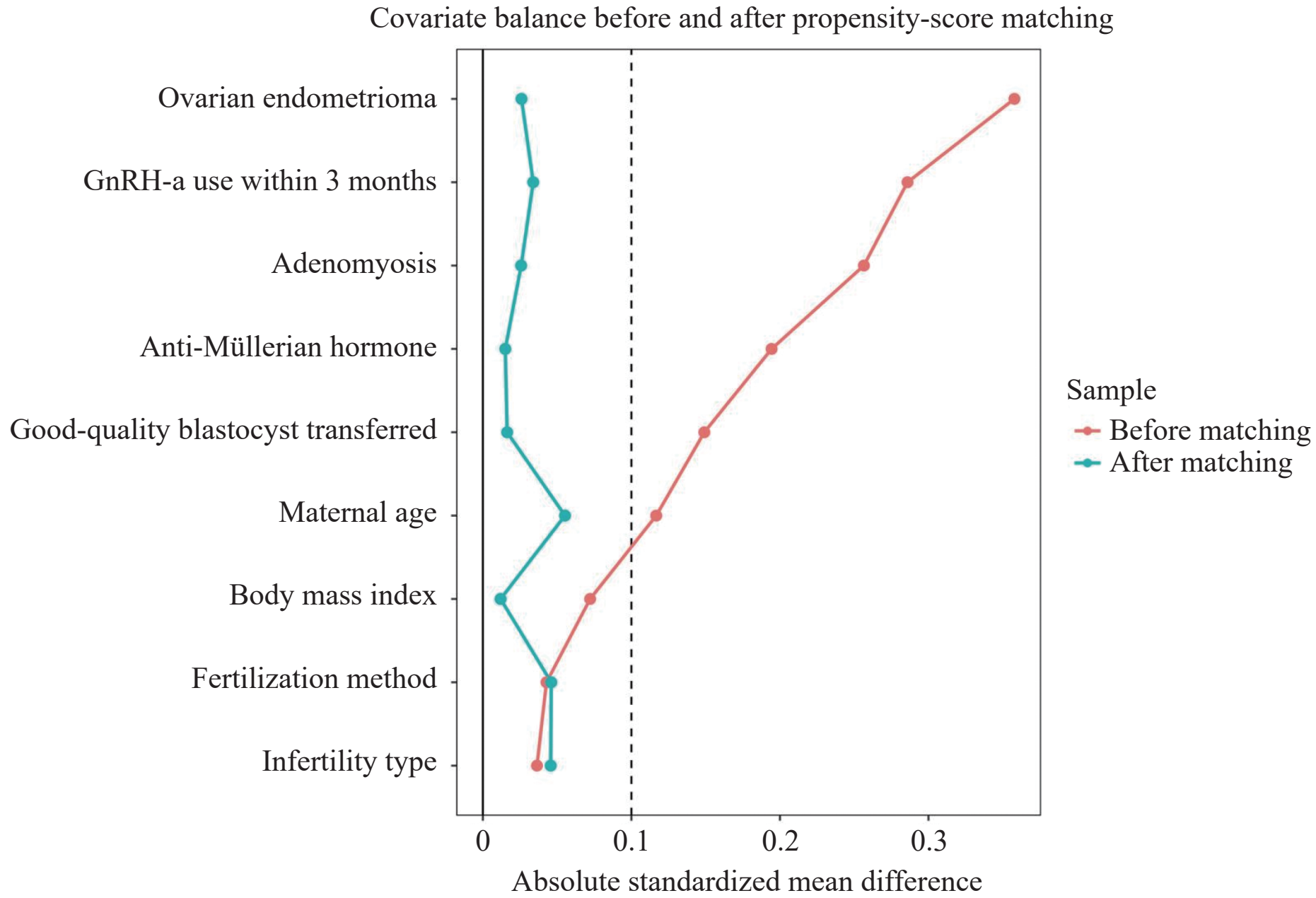


***Supplementary Fig. 1*** **Standardized mean differences before and after propensity score matching.** Absolute standardized mean differences are shown before and after 1∶1 propensity score matching. The dashed vertical line indicates the prespecified balance threshold of 0.10. All covariates had absolute standardized mean differences below 0.10 after matching.

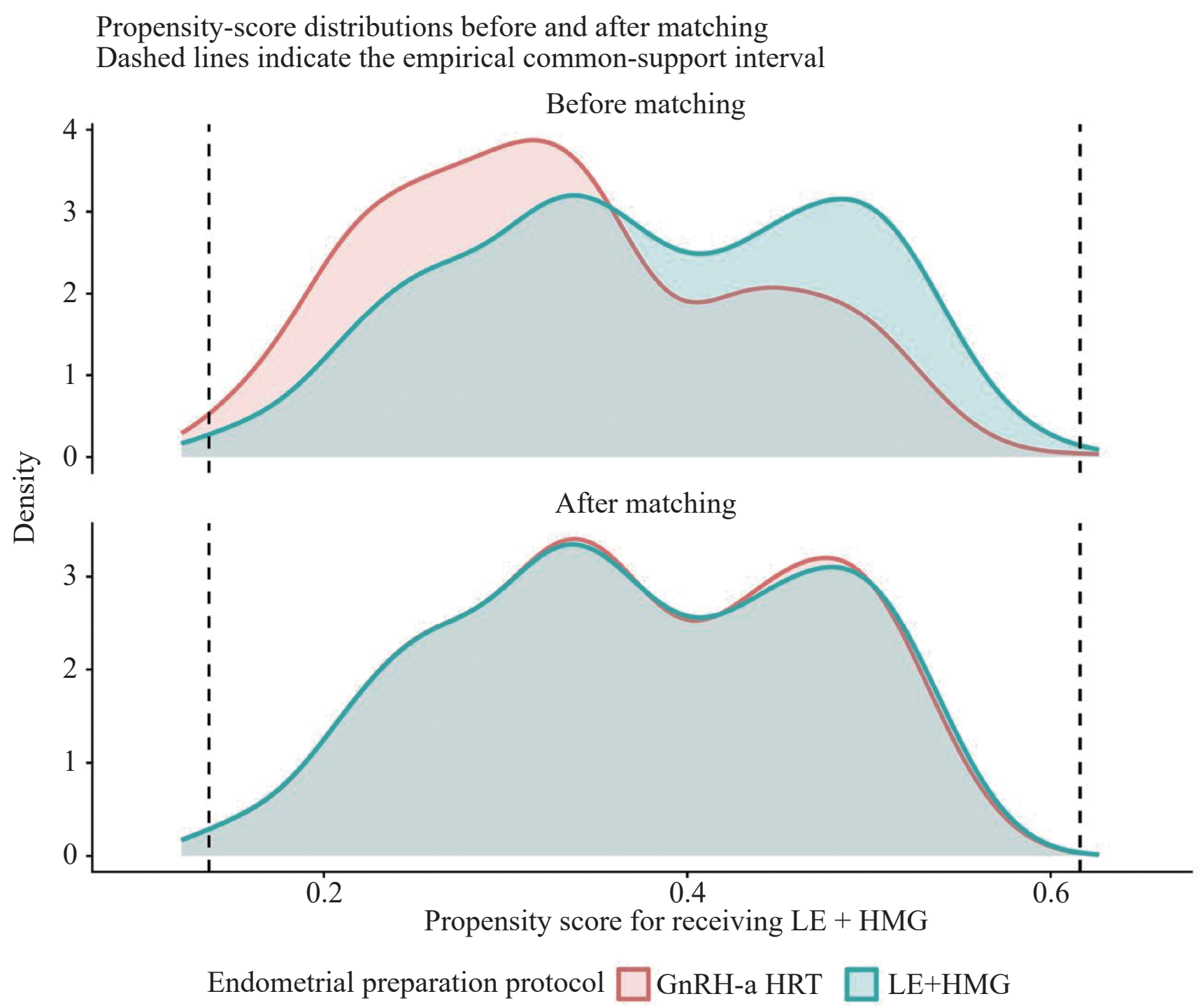


***Supplementary Fig. 2*** **Propensity score distributions before and after matching.** Density plots of the estimated propensity scores in the LE + HMG and GnRH-a HRT groups before and after matching. Dashed vertical lines indicate the empirical common-support interval of 0.137–0.616. Substantial overlap was observed after matching.
